# Allosteric inhibitor remotely modulates the conformation of the orthestric pockets in mutant IDH2/R140Q

**DOI:** 10.1038/s41598-017-16427-w

**Published:** 2017-11-28

**Authors:** Jiao Chen, Jie Yang, Xianqiang Sun, Zhongming Wang, Xiaolan Cheng, Wuguang Lu, Xueting Cai, Chunping Hu, Xu Shen, Peng Cao

**Affiliations:** 1 Key Laboratory of Drug Targets and Drug Leads for Degenerative Diseases, Affiliated Hospital of Integrated Traditional Chinese and Western Medicine, Nanjing University of Chinese Medicine, Nanjing, Jiangsu China; 2 Laboratory of Cellular and Molecular Biology, Jiangsu Province Academy of Traditional Chinese Medicine, Nanjing, Jiangsu China; 3 Pharmaceutical Research Center, School of pharmacy, Guangzhou Medical University, 195 Dongfengxi Road, Guangzhou, China; 4 Division of Theoretical Chemistry and Biology, School of Biotechnology, KTH Royal Institute of Technology, S-106 91 Stockholm, Sweden

## Abstract

Neomorphic mutation R140Q in the metabolic enzyme isocitrate dehydrogenase 2 (IDH2) is found to be a driver mutation in cancers. Recent studies revealed that allosteric inhibitors could selectively inhibit IDH2/R140Q and induce differentiation of TF-1 erythroleukemia and primary human AML cells. However, the allosteric inhibition mechanism is not very clear. Here, we report the results from computational studies that AGI-6780 binds tightly with the divalent cation binding helices at the homodimer interface and prevents the transition of IDH2/R140Q homodimer to a closed conformation that is required for catalysis, resulting in the decrease of the binding free energy of NADPHs. If the allosteric inhibitor is removed, the original open catalytic center of IDH2/R140Q will gradually reorganize to a quasi-closed conformation and the enzymatic activity might recover. Unlike IDH2/R140Q, AGI-6780 locks one monomer of the wild-type IDH2 in an inactive open conformation and the other in a half-closed conformation, which can be used to explain the selectivity of AGI-6780. Our results suggest that conformational changes are the primary contributors to the inhibitory potency of the allosteric inhibitor. Our study will also facilitate the understanding of the inhibitory and selective mechanisms of AG-221 (a promising allosteric inhibitor that has been approved by FDA) for mutant IDH2.

## Introduction

Oncogenic mutations contributing to the metabolic reprogramming are emerging hallmarks of various cancers^1^. Isocitrate dehydrogenases (IDHs) catalyze the conversion of isocitrate to α-ketoglutarate (αKG) in the citric acid cycle, participating in various molecular processes including histone and DNA modifications. Heterozygous point mutations in the active site arginine residues of IDH1 (R132) and IDH2 (R140 and R172) are observed in cancers including low-grade gliomas, secondary glioblastomas, acute myeloid leukemia (AML), angioimmunoblastic T-cell lymphomas, myelodysplastic syndrome (MDS), *etc*.^2–6^. Approximately 10–40% of patients with AML carry mutations in the IDH1/2 gene, making the two proteins promising therapeutic targets in AML^7,8^.

Cancer-associated IDH1/2 mutations lead to the loss of the enzyme’s normal catalytic activity and gain neomorphic activity of reducing αKG to (R)-2-hydroxyglutarate (2-HG), which can be detected at high levels in gliomas and AML patients harboring these mutations^9,10^. 2-HG, structurally similar to αKG, can competitively inhibit αKG-dependent enzymes, such as methylcytosine dioxygenases of the Tet family and histone and DNA demethylases, which regulate the epigenetic state of cells^11–13^. It has been shown that this epigenetic dysregulation promotes a block in cellular differentiation and progression to leukemia^14–17^. Thus, 2-HG is thought to be the main trigger for cancer development in tumors with IDH mutations.

**Figure 1:**
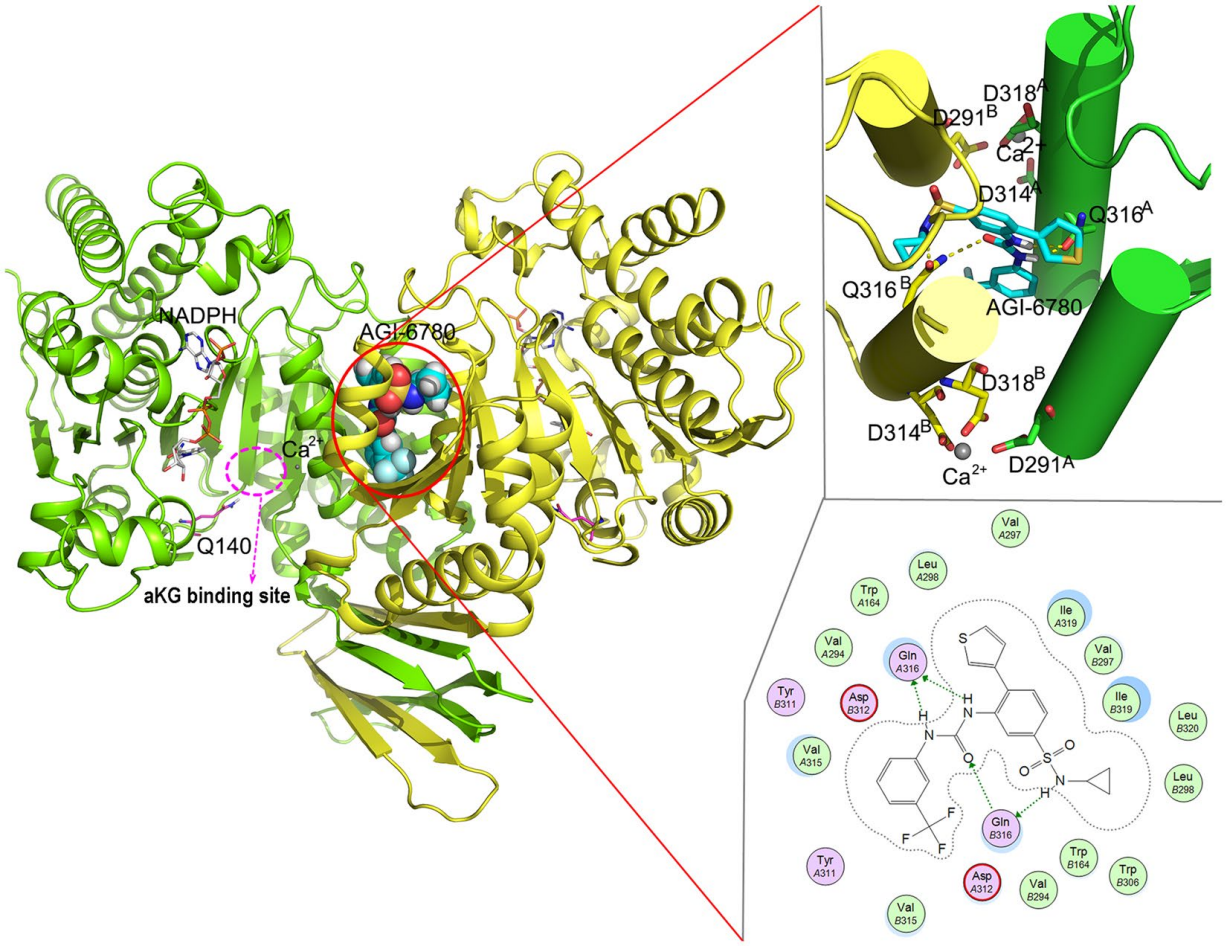
AGI-6780 binds in an allosteric manner at the dimer interface of IDH2/R140Q. The detailed interaction mode is shown in the right panel.

In recent years, IDH mutants have become intriguing targets for cancer therapeutic intervention. A series of inhibitors was reported to have promising preclinical efficacy and early-phase clinical activity in IDH1/2 mutant glioma and AML cells^18–26^. Among them, AGI-6780 is a selective sulfonamide inhibitor of the IDH2/R140Q mutant, with IC_50_ of 23 nM for the homodimeric enzyme while 190 nM for the wild-type IDH2 (defined as IDH2/WT). It reduces intracellular 2-HG level and induces differentiation of TF-1 erythroleukemia and primary human AML cells carrying IDH2/R140Q mutation *in vitro*, providing evidence that inhibition of the mutant IDH2 enzyme can reverse some of the epigenetic changes it induces. Crystal structure reveals that AGI-6780 binds in an allosteric manner at the dimer interface of IDH2/R140Q, which is far from the mutated residue Q140 (Fig. 1), suggesting that the inhibition effect caused by AGI-6780 is rarely dependent on the contact with Q140^19^. But how the binding of AGI-6780 remotely affects the enzymatic activity and the molecular mechanisms of its selectivity for IDH2/R140Q over IDH2/WT remain elusive.

Molecular dynamics (MD) simulations have proved to be an effective approach on such processes^27,28^. Therefore, we carried out a series of MD simulations on the AGI-6780-bound or not bound IDH2/R140Q mutant systems. Combined conformational changes and binding energy analyses, the inhibitory mechanism of the allosteric inhibitor against mutant IDH2 was investigated. Then the effect of the crucial residue Q316 on the binding of AGI-6780 with IDH2/R140Q was studied. To understand the selectivity of AGI-6780 for IDH2/R140Q over IDH2/WT, the dynamics of IDH2/WT in complex with AGI-6780 was characterized and compared with the system of IDH2/R140Q_AGI-6780.

## Methods

### System Preparation

The structures of IDH2/R140Q in complex with the inhibitor AGI-6780 (PDB ID: 4JA8) and the substrate αKG (PDB ID: 5I95) were obtained from the protein data bank. The structures of IDH2/WT and IDH2/R140Q^Q316A^ mutant were built in Discovery Studio 4.1 using the crystal structure 4JA8 as the template. AGI-6780 was copied to the complex structures. All the missing hydrogen atoms were added using the Leap module in Amber 12^29^. Protonation states for the titratable residues were set according to the protein preparation results obtained from the Schrödinger 2015-3 software. Amber ff12SB force field was assigned for the proteins. The structures of AGI-6780 and αKG were optimized with Gaussian 09 program^30^ at the HF/6-31 G* level, and then restrained electrostatic potential (RESP) charges and the general Amber force field (GAFF) were assigned for the optimized structures. The parameters of NADPH and calcium ion were obtained from the Amber parameter database (http://research.bmh.manchester.ac.uk/bryce/amber). The complexes were solvated in a truncated octahedral box of TIP3P water molecules with 10.0 Å buffer along each dimension. Cl^−^ counterions were added to neutralize the systems.

### Molecular dynamics simulations

For each system, energy minimization and MD simulation were performed using the Pmemd module in Amber 12. A two-step energy minimization process based on the steepest descent method followed by the conjugate gradient algorithm was carried out to relieve bad contacts. At the first step, water molecules and counterions were relaxed by restraining the complex with a harmonic constant of 4.0 kcal/mol·Å^−2^. At the second step, the restraint was removed to allow all of the atoms to move freely. After minimization, each system was gently heated from 0 to 300 K in 450 ps at constant volume with a harmonic constant of 10.0 kcal/mol·Å^−2^. Then six steps of constraint equilibrations were carried out at 300 K and 1 bar constant pressure with harmonic constants of 10.0, 8.0, 6.0, 4.0, 2.0 and 1.0 kcal/mol·Å^−2^ respectively. Each step was equilibrated for 2 ns. Finally, a 400 ns MD simulation without any restrictions was performed with a time step of 2 fs. During the MD simulation, all bonds involving hydrogen atoms were constrained using the SHAKE algorithm^31^. The non-bonded cutoff was set to 10.0 Å, and electrostatic interactions were calculated using the particle-mesh Ewald method (PME)^32^. The temperature was controlled using the Langevin thermostat method^33^.

### Data analysis

The cpptraj analysis module within Amber 12 was used for the calculations of root mean square deviation (RMSD), distances and angles. The central structure of the major cluster based on the protein RMSDs of the last 100 ns of each MD trajectory was used for analysis. The direction of motion between two specified states was visualized using the modevectors.py script in PyMOL 1.7.4. Elastic Network Models (ENM) have been successfully used in reproducing fluctuations for proteins of native conformations. PyANM is developed as a cross-platform PyMOL Plugin to visualize ENM. So we adopted pyanm.py script in PyMOL 1.7.4 to visualize the native dynamics of IDH2/R140Q.

Compared with MM/PBSA, MM/GBSA is more suitable in the use of multi-target binding free energy comparisons^34^. Therefore, MM/GBSA of AGI-6780 binding with the enzyme in each system was calculated in Amber 12. A total of 200 snapshots from the 350–400 ns equilibrated dynamics trajectory were extracted, and the MM/GBSA calculation was performed on each snapshot. The binding free energy (ΔG) was computed according to the following equations:^34^

1 $${\rm{\Delta }}{\rm{G}}= < {\rm{\Delta }}{{\rm{G}}}_{{\rm{gas}}} > + < {\rm{\Delta }}{{\rm{G}}}_{{\rm{solv}}} > - < {\rm{T}}{\rm{\Delta }}{\rm{S}} > $$

2 $${\rm{\Delta }}{{\rm{G}}}_{{\rm{gas}}}={{\rm{\Delta }}{\rm{G}}}_{{\rm{elec}}}+{\rm{\Delta }}{{\rm{G}}}_{{\rm{vdW}}}+{\rm{\Delta }}{{\rm{G}}}_{{\rm{int}}}$$

3 $${\rm{\Delta }}{{\rm{G}}}_{{\rm{sol}}}={\rm{\Delta }}{{\rm{G}}}_{{\rm{GB}}}+{\rm{\Delta }}{{\rm{G}}}_{{\rm{np}}}$$

4 $${{\rm{\Delta }}{\rm{G}}}_{{\rm{np}}}={\rm{\gamma }}\times {\rm{SASA}}+{\rm{\beta }}$$

where <…> indicates an average of the energy term. ΔG_gas_ and ΔG_solv_ represent the vacuum and solvation binding free energies, respectively. −TΔS is the entropic contribution, which is not considered in the relative free energy analysis. ΔG_gas_ is composed of intermolecular electrostatic energy (ΔG_elec_), van der Waals energy (ΔG_vdW_), and internal energy (ΔG_int_). ΔG_solv_ includes the electrostatic solvation energy (ΔG_GB_) and the nonpolar solvation energy (ΔG_np_). γ is the surface tension proportionality constant and β is the offset value. The solvent accessible surface area (SASA) was estimated by the MSMS algorithm with a probe radius of 1.4 Å.

## Results and Discussion

### Overview of the structure

In tumor cells harboring IDH mutations, the majority of enzymes would be heterodimeric, with one WT and one mutant monomer^35^. However, no heterodimeric structure of IDH2/R140Q has been determined. So we used the crystal structure of AGI-6780 in complex with IDH2/R140Q homodimer (PDB ID: 4JA8) in the following study. IDHs are NADPH and divalent cation dependent enzymes. The catalytic active site contains binding sites for NADPH, the substrate and a divalent metal cation. The substrate of αKG is absent in the structure of 4JA8. The divalent cation Ca^2+^, which is essential for the enzymatic activity, is chelated with D291 from one monomer and D314, D318 from the other monomer. AGI-6780 doesn’t have direct interaction with Q140, but binds to the allosteric site enclosed within the homodimer interface composed by four helices (residues 290 to 299 and 310 to 322 from each monomer) and two loops (residues 152 to 167 from each monomer), which is distant from the substrate-binding site (Fig. 1). This indicates that direct interactions between the mutated residue and allosteric inhibitor do not account for the inhibitory selectivity. The urea group and amide nitrogen of AGI-6780 makes several direct hydrogen bonding interactions with Q316 in the divalent cation binding helix. Hydrophobic interactions with the surrounding residues W164, V294, V297, L298, Y311, V315, I319, and L320 contribute to AGI-6780’s high inhibitory potency. The dominant hydrophobic pocket suggests larger polar substituents of the inhibitor will be less favorable. Structural rearrangements would be required for the allosteric inhibitor to access to the deeply buried binding site.

### AGI-6780 decreases the binding free energy of NADPHs with IDH2/R140Q

**Figure 2:**
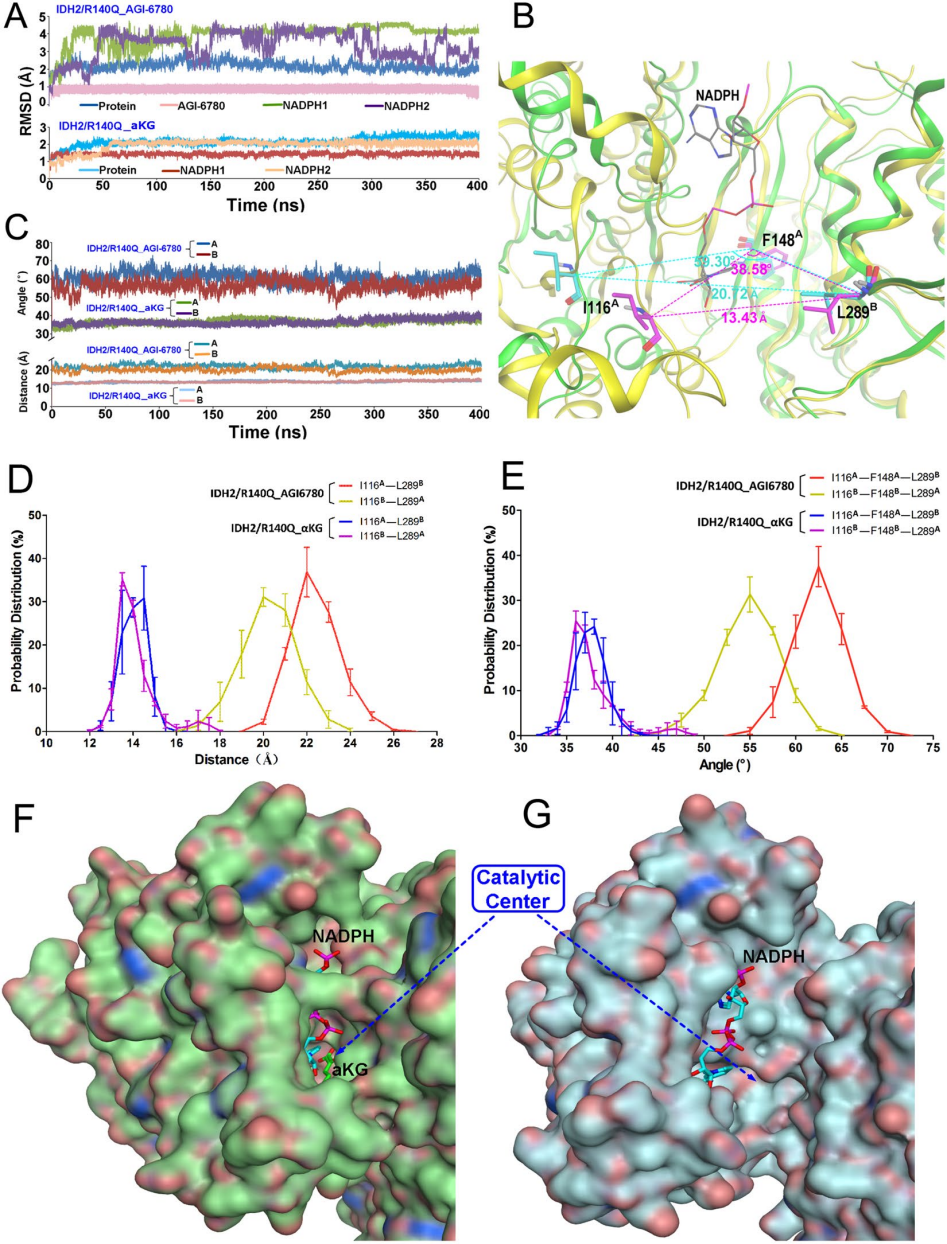
Molecular dynamics (MD) simulations of IDH2/R140Q_αKG and IDH2/R140Q_AGI-6780 systems. (**A**) Evolution of RMSDs during 400 ns MD simulation. (**B**) The active site entrance of IDH2/R140Q. The residues of I116, F148 and L289 are colored purple and cyan respectively in the structures of IDH2/R140Q_αKG (green) and IDH2/R140Q_AGI-6780 (yellow). (**C**) Evolution of the Cα distances of Ile116-Leu289′ and angles of Ile116-Phe148-Leu289′ in monomer **A** and **B** during MD simulation. **D** and **E** represent the probability distributions of Cα distances of Ile116-Leu289′ and angles of Ile116-Phe148-Leu289′. **F** and **G** represent the surface maps around the catalytic center of IDH2/R140Q_αKG and IDH2/R140Q_AGI-6780 respectively.

To understand how AGI-6780 inhibits the neomorphic activity of IDH2/R140Q, MD simulations of IDH2/R140Q_αKG and IDH2/R140Q_AGI-6780 systems were conducted. In our simulations, the protein of both systems reached equilibrium at about 50 ns as indicated from the RMSD values in the simulations (Fig. 2A). The difference is that NADPHs in IDH2/R140Q_AGI-6780 system underwent considerable conformational changes as indicated by their RMSD values, which ranged from 0.3 to 4.4 Å. Comparatively, the evolution of RMSDs of NADPHs in IDH2/R140Q_αKG system was more stable (Fig. 2A), although no experimental values have been reported to compare the binding affinity of NADPHs with the inhibitor-bound or not bound IDH2/R140Q. Dang L *et al*. reported that IDH mutants increase metabolic stress via a reduced NADPH production capacity and gain affinity in a three-order-of-magnitude of NADPH compared with the wild-type enzyme^35^. While in the presence of inhibitors, the IDH-mediated NADPH production capacity of cells harboring IDH mutant was restored to the levels comparable with those of wild-type cells^36^. Accordingly, it can be speculated that the binding affinity between NADPH and the mutant enzyme would be decreased in the presence of inhibitors.

Then we calculated the binding free energy of NADPH with IDH2/R140Q (in the presence or absence of AGI-6780) using the crystal structures in Discovery Studio 4.1 and redock NADPHs into the enzymes. The binding free energy decreased to −509.75 and −646.63 kcal/mol for NADPHs in monomer A and B in the AGI-6780-bound complex, much smaller than that in IDH2/R140Q_αKG complex (−1418.89 and −1382.28 kcal/mol for NADPH^A^ and NADPH^B^). The reDock score of NADPHs also decreased significantly in the AGI-6780-bound complex (Table 1), the tendency of which is in consistent with the binding free energy. Combined with the MD simulation results, we can see that the binding of NADPHs with IDH2/R140Q_AGI-6780 complex is less stable than that with IDH2/R140Q_αKG which is free of inhibitors. The above simulations indicate that AGI-6780 may change the conformation of IDH2/R140Q around the NADPH binding site and affects the binding of NADPHs with the enzyme. For an NADPH-dependent enzyme, decreased binding free energy of NADPH might contribute to the loss of the enzyme’s catalytic ability inhibited by AGI-6780.

**Table 1:** The calculated binding free energy and reDock scores of NADPHs with IDH2/R140Q in the presence/absence of AGI-6780.

Systems	NADPH	Binding free energy (kcal/mol)	reDock Score
IDH2/R140Q_AGI6780	NADPH^A^	−509.75	−12.99
	NADPH^B^	−646.63	−12.60
IDH2/R140Q_αKG	NADPH^A^	−1418.89	−20.60
	NADPH^B^	−1382.28	−19.10

### AGI-6780 locks the IDH2/R140Q dimer in an open inactive conformation

AGI-6780-bound enzyme exhibits pronounced movement of one monomer with respect to the other. The width of the active site entrance (defined as the distance between I116 of one monomer and L289 of the adjacent monomer (I116-L289′), and the angle of I116, F148 of one monomer and L289 of the adjacent monomer (I116-F148-L289′)) of IDH2/R140Q_αKG is much narrower than IDH2/R140Q_AGI-6780 (Fig. 2B). Correspondingly, the evolution of the distance and angle in both systems during the simulation are shown in Fig. 2C. In IDH2/R140Q_AGI-6780 system, the average distances of I116-L289′ during 300–400 ns period are 22.01 Å and 19.34 Å, and the average angles of I116-F148-L289′ are 60.77° and 54.43° respectively for A and B monomer (Table 2). Compared with IDH2/R140Q_αKG, these values are much larger (Fig. 2C, Table 2), indicating much wider active site pockets in IDH2/R140Q_AGI-6780. To quantify the uncertainties and ensure the reliability of our conclusion, we repeated the simulations (each 400 ns) for the systems of IDH2/R140Q_AGI6780 and IDH2/R140Q_αKG. Bootstrapping method^37,38^ was also carried out to evaluate the statistical error for the width of the active site entrance. The probability distributions of the distances of I116-L289′ and angles of I116-F148-L289′ are shown in Fig. 2D and E, with quantified uncertainties/errors. We can see that αKG binding can induce conformational changes of the active site and inter-monomer motion of IDH2/R140Q which is free of AGI-6780. To be more intuitive, the surface maps of IDH2/R140Q_αKG and IDH2/R140Q_AGI-6780 are presented in Fig. 2F and G, respectively. Obviously, the catalytic active site of IDH2/R140Q in complexed with AGI-6780 is much wider than that with αKG. These results demonstrate that IDH2/R140Q_αKG adopts a compact closed conformation while AGI-6780 allosterically stabilizes the open homodimer conformation of IDH2/R140Q.

**Table 2:** The Cα distances of Ile116-Leu289′ and angles of Ile116-Phe148-Leu289′.

System	Distance		Angle	
	I116^A^-289^B^ (Å)	I116^B^-L289^A^ (Å)	I116^A^-F148^A^-L289^B^ (°)	I116^B^-F148^B^-L289^A^ (°)
IDH2/R140Q_AGI6780^a^	22.24	20.37	61.10	56.30
IDH2/R140Q_AGI6780^b^	22.01 ± 0.96	19.34 ± 0.97	60.77 ± 2.56	54.43 ± 2.98
IDH2/R140Q^b^	14.62 ± 0.68	17.95 ± 0.85	40.34 ± 2.05	48.66 ± 2.24
IDH2/R140Q_αKG^c^	12.54	12.54	34.40	34.40
IDH2/R140Q_αKG^b^	14.04 ± 0.33	14.95 ± 1.07	37.41 ± 1.01	40.56 ± 2.98
IDH2/R140Q^Q316A^_AGI6780^b^	20.52 ± 0.76	18.24 ± 0.77	55.76 ± 2.28	49.45 ± 2.33
WT_AGI6780^b^	18.04 ± 0.70	22.75 ± 1.43	49.59 ± 1.81	62.08 ± 3.64

^a^Crystal structure of IDH2/R140Q_AGI6780 (PDB: 4JA8); ^b^MD simulation systems, the average distances and angles were calculated from the 300–400 ns trajectory; ^c^Crystal structure of IDH2/R140Q_αKG (PDB: 5I95).

AGI-6780 binds tightly with the four helices (including the divalent cation binding helix) at the homodimer interface (Fig. 1) and prevents IDH2/R140Q from any substantial changes. As the enzymatic activity requires a conformational transition from an open to a closed conformation upon αKG binding^39^, AGI-6780 locks the IDH2/R140Q dimer in an open conformation incompatible with catalysis and inhibits the transformation of αKG to 2-HG. Moreover, monomer A and B of IDH2/R140Q_AGI-6780 exhibit a little difference in the conformation of the substrate-binding site. The distance of I116-L289′ and angle of I116-F148-L289′ in monomer A are larger than that in monomer B (Fig. 2C, Table 2), so monomer A of IDH2/R140Q_AGI-6780 adopts a more open conformation than monomer B.

**Figure 3:**
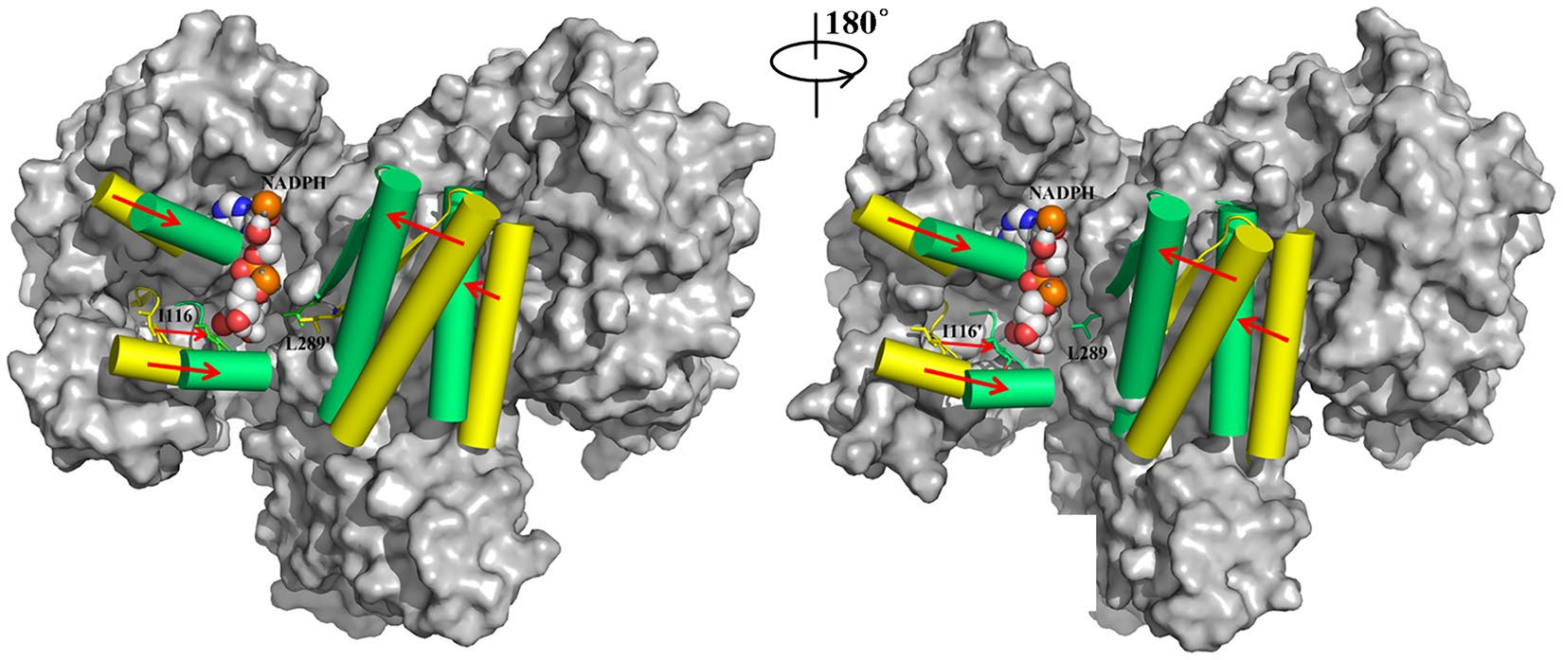
Structural superposition of IDH2/R140Q_AGI-6780 (open conformation, colored yellow) and IDH2/R140Q_αKG (closed conformation, colored green) after 400 ns MD simulation.

To compare the open and closed conformations, the structure of IDH2/R140Q_αKG and IDH2/R140Q_AGI-6780 after 400 ns MD simulation were superposed (Fig. 3). From the conformation of IDH2/R140Q_AGI-6780 to IDH2/R140Q_αKG, the α-helices of the protein move significantly forward to the catalytic centers. The clasp domain (which interlocks the two monomers) in the AGI-6780-bound structure is also displaced in order to adapt to the overall conformational changes, compared with its position in the αKG-bound structure (Fig. S1). Therefore, we hypothesize that if AGI-6780 is removed, the inactive open catalytic center of IDH2/R140Q will reorganize to a closed conformation, and then the neomorphic activity of IDH2/R140Q will recover.

### IDH2/R140Q reorganized to a quasi-closed conformation when AGI-6780 is removed

**Figure 4:**
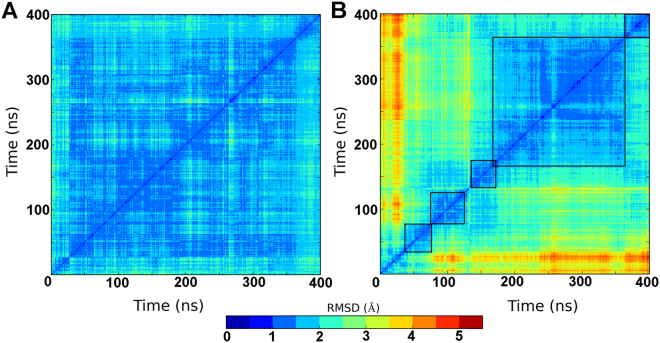
RMSD matrices of IDH2/R140Q_AGI-6780 (**A**) and IDH2/R140Q (**B**) systems during MD simulation.

To test the above hypothesis, AGI-6780 was removed from the system of IDH2/R140Q_AGI-6780 and the remaining complex (defined as a new system: IDH2/R140Q) was subjected to a 400 ns MD simulation. From the RMSD matrices, we can see that after removed AGI-6780, the protein of IDH2/R140Q underwent substantial conformational changes, mainly five clusters (Fig. 4). The width of the active site pockets become narrower, with the average distances of I116-L289′ 14.62 Å and 17.95 Å, and the average angles of I116-F148-L289′ 40.34° and 48.66° respectively for monomer A and B during the last 100 ns (Table 2). These values are much smaller than that in the system of IDH2/R140Q_AGI-6780, but a little bigger than that in IDH2/R140Q_αKG. This indicates that the original open catalytic center of IDH2/R140Q was gradually reorganized to a quasi-closed conformation. Substrate binding would induce the enzyme to a closed conformation, however, due to the absence of αKG in this system, IDH2/R140Q stayed at the quasi-closed conformation after equilibrium in the molecular dynamics simulation. These results confirm that the neomorphic activity of IDH2/R140Q will recover if AGI-6780 is removed from the structure.

Nevertheless, structural comparison suggested that the open conformation of IDH2/R140Q_AGI-6780 is analogous to that observed for the cytosolic IDH1 with the absence of substrate molecules^19^, which seems to have discrepancy from our simulation results. Then the intrinsic dynamics of IDH2/R140Q was reproduced using PyANM (Supplementary Data, movie 1). We can see that the enzyme without substrates can fluctuate from an open conformation to a closed conformation. Thus it is speculated that the crystal open conformation of IDH1 with the absence of substrates might be a transient snapshot. Therefore, it is not contradictory with our simulation results.

### Q316 plays a crucial role in the binding energy of AGI-6780 with IDH2/R140Q

**Figure 5:**
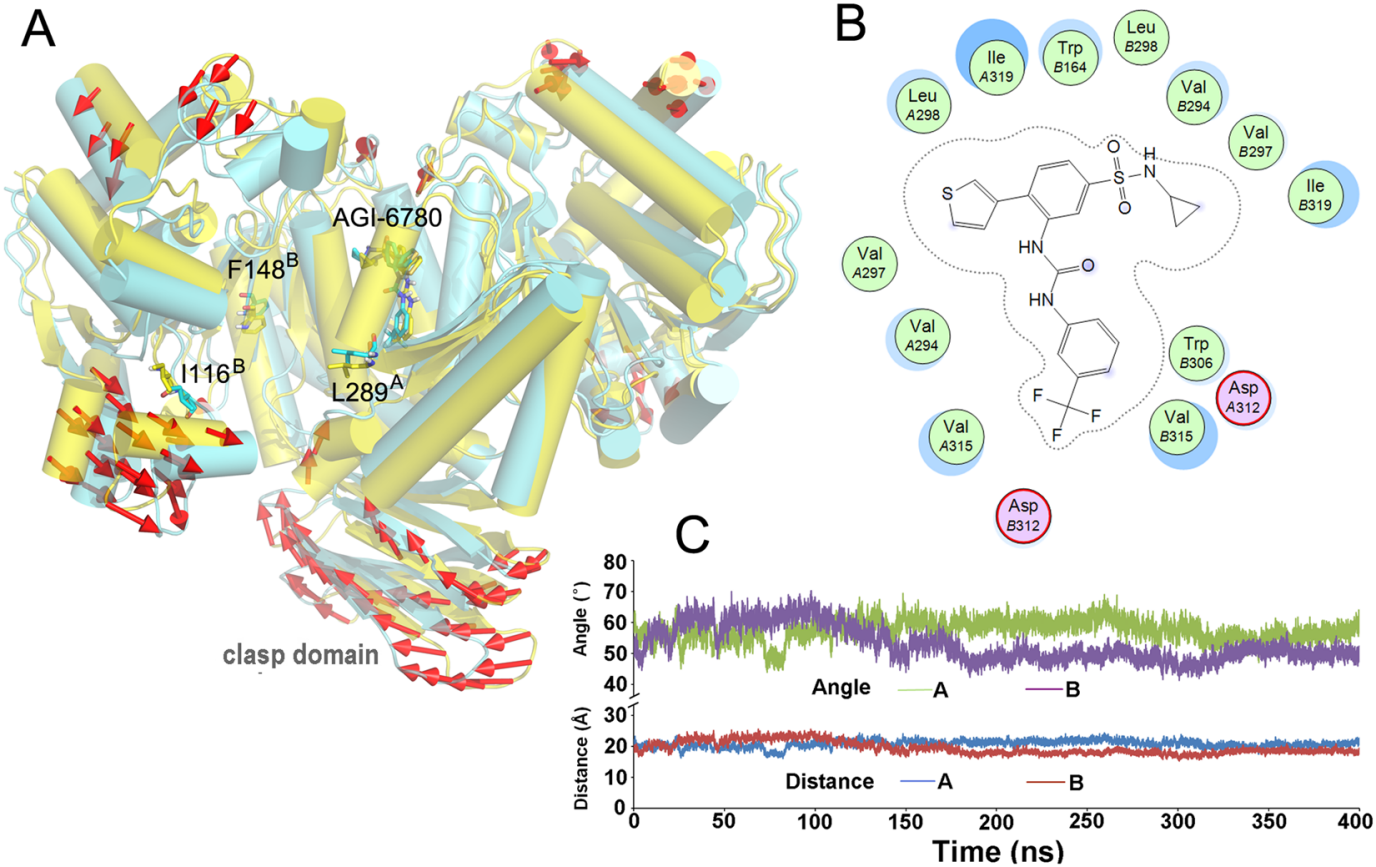
(**A**) The direction of motion between IDH2/R140Q_AGI-6780 (yellow) and IDH2/R140Q^Q316A^_AGI-6780 (cyan) after 400 ns simulation. (**B**) Detailed interaction mode of AGI-6780 with IDH2/R140Q^Q316A^. (**C**) Evolution of the Cα distances of Ile116-Leu289′ and angles of Ile116-Phe148-Leu289′ in the system of IDH2/R140Q^Q316A^_AGI-6780 during MD simulation.

AGI-6780 makes several direct hydrogen bonding interactions with Q316 of IDH2/R140Q. To investigate the effect of Q316 on the affinity of AGI-6780 interacting with IDH2/R140Q, virtual mutant of IDH2/R140Q^Q316A^ was built and a subsequent 400 ns MD simulation was conducted. The direction of motion between IDH2/R140Q_AGI-6780 and IDH2/R140Q^Q316A^_AGI-6780 after 400 ns MD simulation is demonstrated in Fig. 5A. From the conformation of IDH2/R140Q_AGI-6780 to IDH2/R140Q^Q316A^_AGI-6780, there is a slight change in the catalytic center and the pockets become a little narrower indicated by the distances of I116-L289′ and angles of I116-F148-L289′ (Table 2 and Fig. 5A,C). The catalytic domain and clasp domain of IDH2/R140Q^Q316A^ is reorganized (Fig. 5A). The hydrogen bonding interactions disappear when Gln is mutated to Ala, and hydrophobic interactions are dominant in the interactions between AGI-6780 and IDH2/R140Q^Q316A^ (Fig. 5B).

The binding free energy of MM/GBSA is frequently used in the comparison of multi-target binding free energy. So in this study, the MM/GBSAs of AGI-6780 binding with different systems were calculated. From the results we can see that the binding free energy of AGI-6780 with IDH2/R140Q^Q316A^ decreases to −47.08 kcal/mol, much lower than that with IDH2/R140Q (−62.37 kcal/mol, Table 3). This indicates that Q316 is crucial in the binding of AGI-6780 with IDH2/R140Q.

**Table 3:** The binding free energy (MM/GBSA) of AGI-6780 with different IDH2 systems.

Systems	IDH2/R140Q_AGI-6780	IDH2/R140Q^Q316A^_AGI-6780	IDH2/WT_AGI-6780
MM/GBSA (kcal/mol)	−62.37 ± 3.07	−47.08 ± 2.75	−62.25 ± 3.26

### AGI-6780 locks one monomer of IDH2/WT in an inactive open conformation and the other in a half-closed conformation

AGI-6780 exhibits excellent potency against the neomorphic activity of the mutant IDH2/R140Q homodimer enzyme with IC_50_ of 23 nM, while less potency against the normal oxidative decarboxylation activity of IDH2/WT with IC_50_ of 190 nM. Because of the importance of IDH2/WT in primary metabolism, selective inhibition of the mutant enzyme over the wild-type is a critical issue, however, the mechanistic basis for this selectivity remains an important unsolved question.

**Figure 6:**
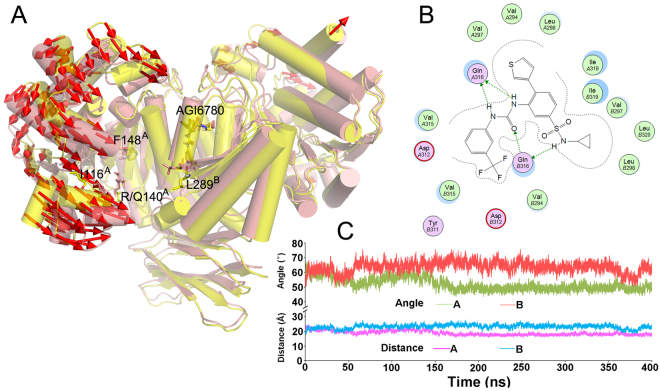
(**A**) The direction of motion between IDH2/R140Q_AGI-6780 (yellow) and IDH2/WT_AGI-6780 (pink) after 400 ns simulation. (**B**) Detailed interaction mode of AGI-6780 with IDH2/WT. (**C**) Evolution of the Cα distances of Ile116-Leu289′ and angles of Ile116-Phe148-Leu289′ in the system of IDH2/WT_AGI-6780 during MD simulation.

In this study, MD simulation of IDH2/WT_AGI-6780 was performed in order to investigate the above question. The model of IDH2/WT_AGI-6780 was built based on the structure of IDH2/R140Q_AGI-6780, and a subsequent 400 ns MD simulation was conducted. After simulation, the catalytic pocket of monomer A in IDH2/WT exhibits a significant closing tendency compared with the structure of IDH2/R140Q_AGI-6780 (Fig. 6A), with the average distance of I116-L289′ 18.04 Å and angle of I116-F148-L289′ 49.59° (the corresponding values are 22.01 Å and 60.77° in the system of IDH2/R140Q_AGI-6780) during the last 100 ns trajectory (Table 2, Fig. 3C). While for monomer B, the width of the active site entrance becomes a little broader in IDH2/WT unexpectedly, with the average distance of I116-L289′ 22.75 Å and angle of I116-F148-L289′ 62.08° (the corresponding values are 19.34 Å and 54.43° in the system of IDH2/R140Q_AGI-6780) (Table 2, Fig. 3C). Additionally, little difference is observed between the interaction mode of AGI-6780 with the enzymes of IDH2/WT (Fig. 6B) and IDH2/R140Q (Fig. 1), and the binding free energy (MM/GBSA) of AGI-6780 with IDH2/WT is −62.25 kcal/mol, similar to that with IDH2/R140Q (−62.37 kcal/mol, Table 3). This indicates that the changed conformation did not affect the binding of AGI-6780 with the enzyme of IDH2/WT. However, the apparent activity of a ligand is affected not only by its affinity with the protein^40,41^. Different ligands with similar binding energy might exhibit an opposite effect because they induce different conformational changes in the protein (*eg*. agonist and antagonist). In this study, AGI-6780 can lock one monomer of IDH2/WT in an inactive open conformation, meanwhile, the other monomer in a half-closed conformation. We speculate that the half-closed conformation monomer may have some residual oxidative decarboxylation activity, which leads to the less inhibitory potency of AGI-6780 against IDH2/WT.

## Conclusion

AML is a highly malignant cancer with poor prognosis and novel treatment strategies to improve prognosis are urgently needed. Mutant IDH proteins are becoming promising therapeutic targets for the treatment of AML and other related cancers. AGI-6780 is confirmed to be the first reported selective inhibitor of the IDH2/R140Q mutant. It binds at the dimer interface of IDH2/R140Q in an allosteric manner. To understand how AGI-6780 affects the enzymatic activity and the molecular mechanisms of its selectivity for IDH2/R140Q over IDH2/WT intuitively, MD simulations of different systems were conducted and analyzed. From the system of IDH2/R140Q_αKG, we can see that the substrate of αKG can induce the mutated enzyme to a closed conformation that is required for catalysis. AGI-6780 binds tightly with the divalent cation-binding helices at the homodimer interface and locks the IDH2/R140Q dimer in an open conformation, leading to the inactivation of the enzyme. We found that the binding of AGI-6780 with IDH2/R140Q affects the binding of NADPH cofactor, which may also contribute to the impaired activity of the enzyme. Q316 is crucial in the binding of AGI-6780 with IDH2/R140Q, if mutated to Ala, the binding energy will decrease significantly. If removed the allosteric inhibitor, the original open catalytic center of IDH2/R140Q will gradually reorganize to a quasi-closed conformation and the neomorphic enzyme activity might recover. To understand the mechanism of selectivity, the binding of AGI-6780 with IDH2/WT was investigated. AGI-6780 locks one monomer of IDH2/WT in an inactive open conformation and the other in a half-closed conformation. The half-closed conformation monomer is speculated to have some residual oxidative decarboxylation activity. These different conformational changes might be the primary cause of the less inhibitory potency of AGI-6780 against IDH2/WT.

Targeting the mutant IDH2 enzyme, the promising inhibitor AG-221 (enasidenib) has been granted orphan drug for the treatment of AML by FDA. Clinical trials demonstrate that AG-221 produced an overall response rate of 40.3% in relapsed/refractory AML patients with mutant IDH2, including some complete remissions^20,42–44^. AG-221 is also a selective allosteric inhibitor of IDH2/R140Q with similar interaction mode of AGI-6780. Therefore, this study will facilitate the understanding of the inhibitory and selective mechanisms of AG-221 for mutant IDH2 as well.

## Electronic supplementary material

Supplementary data

The intrinsic dynamics of IDH2/R140Q reproduced by PyANM
