# Supplementary material for: Allosteric inhibitor remotely modulates the conformation of the orthestric pockets in mutant IDH2/R140Q
Source: Sci Rep. 2017 Nov 28;7:16458. doi: 10.1038/s41598-017-16427-w (PMC5705638; doi:10.1038/s41598-017-16427-w)
Supplement: Supplementary file 1 — Supplementary data [file 41598_2017_16427_MOESM1_ESM.docx]

**Allosteric inhibitor remotely modulates the conformation of the orthestric pockets in mutant IDH2/R140Q**

Jiao Chen^1,2^, Jie Yang^1,2^, Xianqiang Sun^3,4^, Zhongming Wang^1,2^, Xiaolan Cheng^1,2^, Wuguang Lu^1,2^, Xueting Cai^1,2^, Chunping Hu^1,2^, Xu Shen^1^, Peng Cao^1,2^*

1. Key Laboratory of Drug Targets and Drug Leads for Degenerative Diseases, Affiliated Hospital of Integrated Traditional Chinese and Western Medicine, Nanjing University of Chinese Medicine, Nanjing, Jiangsu, China.

2. Laboratory of Cellular and Molecular Biology, Jiangsu Province Academy of Traditional Chinese Medicine, Nanjing, Jiangsu, China.

3. Pharmaceutical Research Center, School of pharmacy, Guangzhou Medical University, 195 Dongfengxi Road, Guangzhou, China.

4. Division of Theoretical Chemistry and Biology, School of Biotechnology, KTH Royal Institute of Technology, S-106 91 Stockholm, Sweden.

*Corresponding author: Peng Cao, E-mail: [pcao79@yahoo.com](mailto:pcao79@yahoo.com), Nanjing University of Chinese Medicine, 100#, Shizi Street, Hongshan Road, Nanjing, Jiangsu, China. Phone/Fax: 8625-85608666.


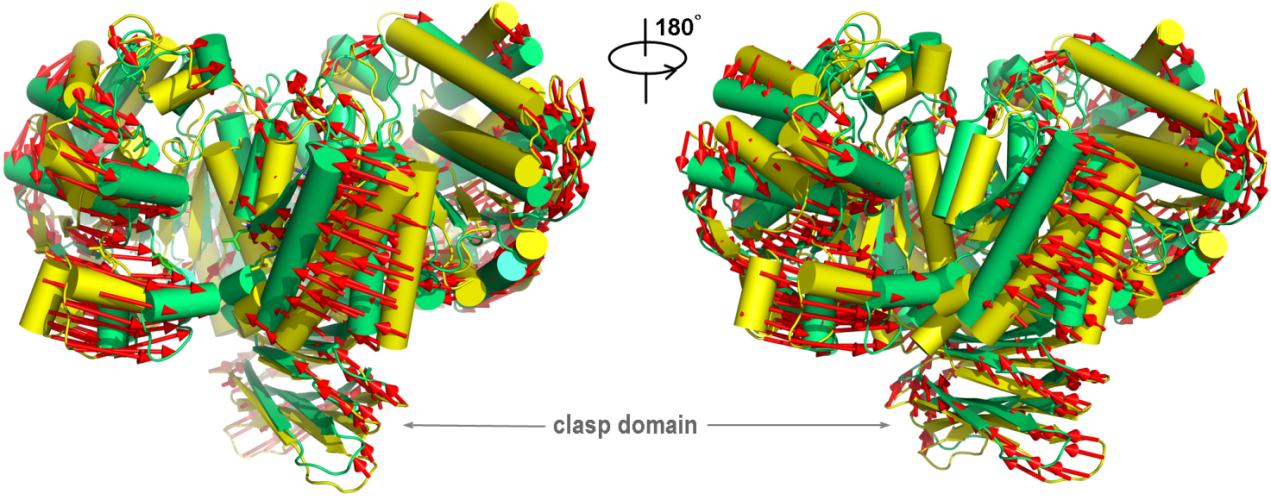


Fig. S1 The direction of motion from IDH2/R140Q_AGI-6780 (yellow) to IDH2/R140Q_αKG (green) after 400 ns simulation.
